# Single-Molecule FRET: A Tool to Characterize DNA Nanostructures

**DOI:** 10.3389/fmolb.2022.835617

**Published:** 2022-03-07

**Authors:** Nibedita Pal

**Affiliations:** Single Molecule Biophysics Lab, Department of Biology, Indian Institute of Science Education and Research Tirupati, Tirupati, India

**Keywords:** DNA nanostructures, DNA nanodevice, real-time detection, SmFRET, conformational dynamics

## Abstract

DNA nanostructures often involve temporally evolving spatial features. Tracking these temporal behaviors in real time requires sophisticated experimental methods with sufficiently high spatial and temporal resolution. Among the several strategies developed for this purpose, single-molecule FRET (smFRET) offers avenues to observe the structural rearrangement or locomotion of DNA nanostructures in real time and quantitatively measure the kinetics as well at the single nanostructure level. In this mini review, we discuss a few applications of smFRET-based techniques to study DNA nanostructures. These examples exemplify how smFRET signals not only have played an important role in the characterization of the nanostructures but also often have helped to improve the design and overall performance of the nanostructures and the devices designed from those structures. Overall, this review consolidates the potential of smFRET in providing crucial quantitative information on structure–function relations in DNA nanostructures.

## Introduction

Programmability, self-assembly, biocompatibility, and easy tailoring of DNA compared to other biopolymers have established it as an excellent building material for nanostructures. In addition to being a structural module, DNA simultaneously acts as a functional element in the nanostructure. DNA nanostructures can be broadly divided into two classes: static and dynamic DNA nanostructures. Dynamic DNA nanostructures primarily function by undergoing controlled structural rearrangement or motion, thus building nanoscale devices. These dynamic DNA nanostructures have been used in a range of applications, from therapeutics (Bujold et al., 2016), drug delivery (Zhang et al., 2014), DNA-based logic gates (Pei et al., 2012) to robotics (Lund et al., 2010), etc. The regulations of the controlled motion mainly include toehold-mediated DNA strand displacement (Goodman et al., 2008; Zhang and Georg, 2011) and application of external stimuli, for example, ions (Mao et al., 1999), light (Yeo et al., 2017), and pH (Liu et al., 2006). Dynamic DNA nanostructures can also be produced by combining self-assembly of static components with reconfigurable modules, which undergo post-assembly conformational changes such as DNA nanobox (Andersen et al., 2009), DNA tweezers (Zhou et al., 2012), DNA walkers (Shin and Pierce, 2004), and reconfigurable origami (Zhang et al., 2012).

**FIGURE 1:**
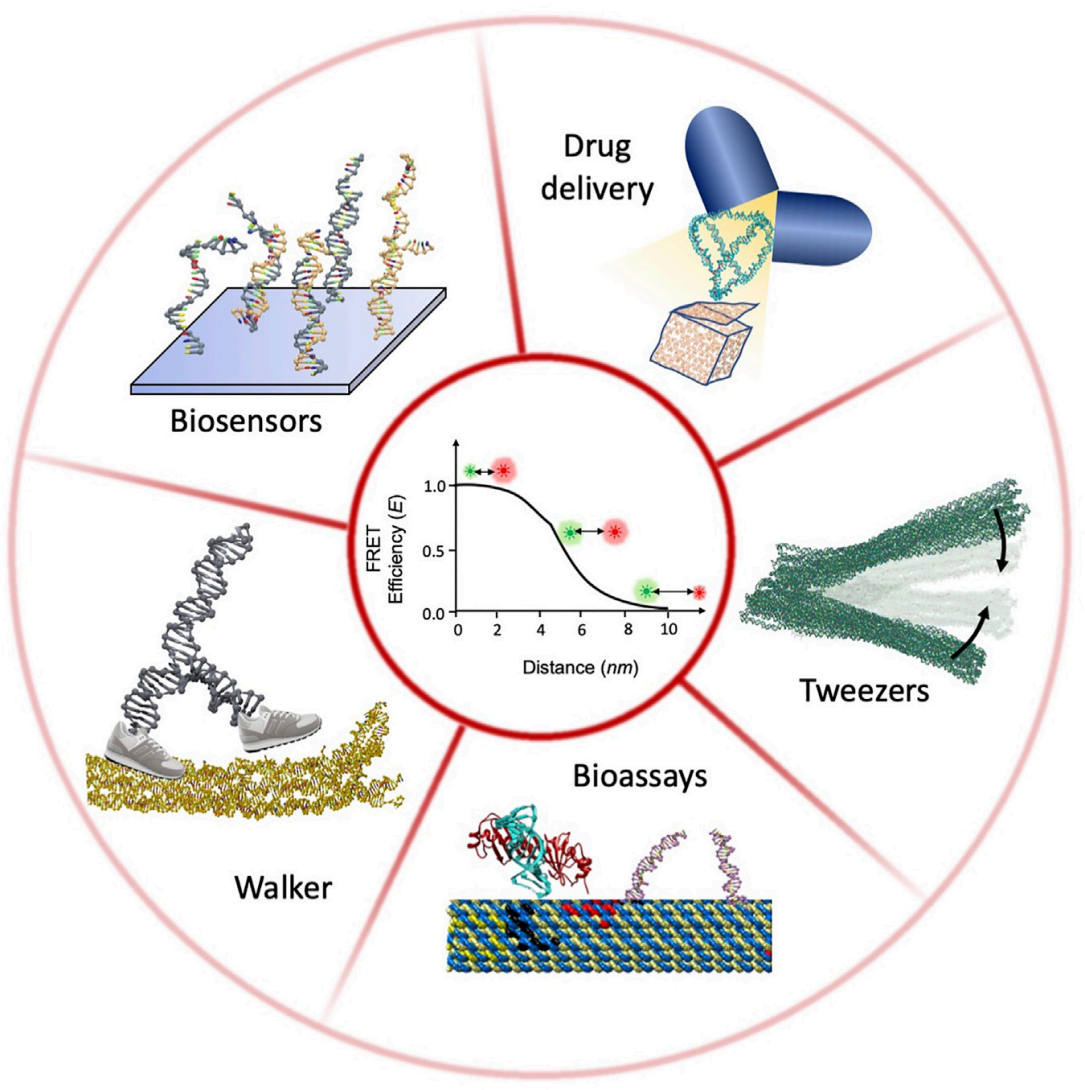
Schematic illustration of DNA nanostructure characterization using smFRET.

Analytical tools such as atomic force microscopy (AFM), transmission electron microscopy (TEM), and cryogenic electron microscope (Cryo-EM) traditionally have been employed to confirm the structural integrity of these nanostructures. However, real-time monitoring of the dynamic DNA nanostructures during their course of action is challenging. The single-molecule Förster resonance energy transfer (smFRET) technique has the capability of receiving real-time structural and functional information of these DNA-made nanostructures. smFRET leverages the spectroscopic phenomenon of distance-dependent non-radiative energy transfer from an excited donor fluorophore to an acceptor fluorophore in the ground state. Thus, it directly reports any change in shape and its time evolution. Additionally, single-molecule methods reveal the structural heterogeneity among the nanostructures, rarely occurring mechanistic steps, and faulty or non-functional nanostructures. smFRET has been increasingly used in diverse characterization of DNA nanostructures and DNA-based nanodevices, such as quality checking, conformational transition, actuation, and operation. These characterizations are crucial for the optimization of the nanostructure and the device constructed from it. The purpose of this mini review is to highlight the recent studies that have utilized smFRET to characterize and optimize DNA nanostructures and DNA nanostructure-based devices. Among those applications, we limit our review to DNA walkers, DNA nanostructure-based drug delivery vehicles, DNA tweezers as a prototype of DNA nanodevices, biosensors, and study of bioassays (Figure 1). These examples demonstrate how smFRET data have been proven beneficial for redesigning or refining the nanostructures for optimum performances by providing high spatial and temporal resolution.

## Real-Time Monitoring of Conformation Transition Using smFRET

FRET measurements start with engineering the system under investigation with two fluorophores (donor and acceptor fluorophores) at precise and judiciously chosen locations. A suitable donor–acceptor pair requires close spatial proximity between them (typically within 1–10 nm), a good spectral overlap between the emission spectrum of the donor and absorption spectrum of the acceptor, and a sufficiently long donor fluorescence lifetime. As the non-radiative energy transfer from the excited donor fluorophore to the acceptor fluorophore in the ground state changes in a distance-dependent manner, any change or rearrangement in the structure will be reflected in the altered emission from both donor and acceptor fluorophores. With proper calibration, the efficiency of energy transfer (*E*) essentially reports nanoscale dimension. Thus, FRET is often described as a spectroscopic ruler. The time trajectories of *E* provide information of the conformational dynamics at nanometer length scale and the effect of external stimuli on it.

**FIGURE 2:**
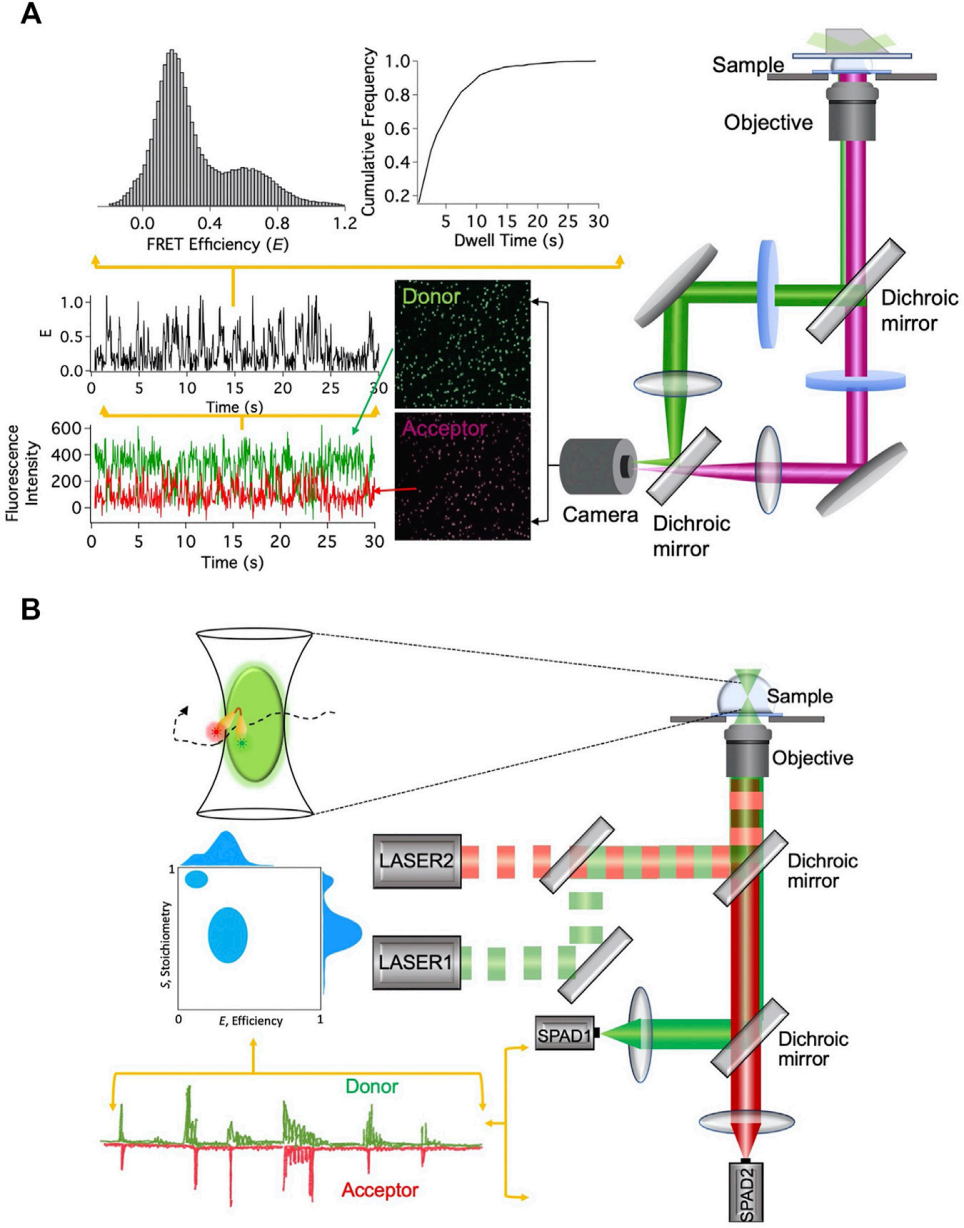
smFRET-based techniques. **(A)** Setup for immobilization-based experiments and data analysis steps. **(B)** Setup for diffusion-based experiments and data analysis steps.

smFRET signals can be acquired after immobilizing the fluorescently labeled nanostructures or during their free diffusion (Figure 2). In wide-field total internal reflection fluorescence microscopy (TIRFM)-based smFRET, the system of interest is immobilized on a surface, typically on a functionalized quartz surface. With significantly high time resolution offered by modern scientific CMOS cameras, simultaneous observation of hundreds of individual molecules in action for a longer period of time has become possible (Ray et al., 2021). As the distance between the donor and the acceptor fluorophores changes, the fluorescence intensities from them change in an anticorrelated fashion. The FRET efficiency *E* trajectory is calculated from the fluorescence intensity trajectories. The dwell times at different FRET states are extracted from the FRET efficiency trajectories and cumulative frequency distribution of the dwell times is constructed. By fitting the cumulative frequency distribution of the dwell time with a proper model, one can estimate the number of interconverting states in the system and the associated kinetic rates (Valero et al., 2018; Ray et al., 2021). Different conformational states are also distinguished from the histograms of dwell times and FRET efficiencies (Figure 2A).

Unlike the TIRFM-based smFRET technique, in diffusion-based single-molecule technique, fluctuations in the fluorescence intensity over time are analyzed, while fluorescently labeled molecules diffuse in and out of the limited observation volume. Diffusion-based smFRET-ALEX (alternating laser excitation) employs two lasers directly exciting the donor and acceptor fluorophores in an alternating fashion (Lee et al., 2005). The fluorescence bursts from the fluorophores produced during their transits through the confocal volume are analyzed for two observables, *E* and the stoichiometry between donor and acceptor fluorophores (*S*). The 2D histogram between *E* and *S* presents and quantifies donor and acceptor stoichiometry and the distance between them (Figure 2B). It enables us to eliminate the contributions from the incompletely labeled molecules and consider only molecules that are labeled with both donor and acceptor fluorophores. This is particularly useful to distinguish only donor-labeled molecules from both donor- and acceptor-labeled molecules with low FRET efficiency. Although both species will exhibit lower *E*, they can be distinctly distinguished from *S* values.

Among various advantages of single-molecule detection technique, one is that it removes the requirement of synchronizing the function of all the structures under investigation. This becomes extremely useful to observe nanodevices during their operation because synchronization is quickly lost after a few initial instances. Furthermore, the construction of the probability distribution of FRET efficiency *E* directly reflects the structural heterogeneity (if there is any) among the nanostructures. This becomes increasingly important in order to separate the faulty or floppy nanostructures from the correct ones. The strength of the smFRET technique has been reflected in the explosive growth in its application in diverse fields in recent times.

## Characterization of DNA Nanostructures Using smFRET

### Observing DNA Walker at Work

Inspired by the biological motor proteins, DNA walker is one of the most sophisticated and fascinating DNA nanodevices designed so far. With the recent development in the design strategy, DNA walkers find attractive applications in molecular computing (Dannenberg et al., 2015), signal transduction, and biosensing (Wang et al., 2017). The large variety of mechanisms employed by the walker to take the next step includes strand displacement/exchange mechanism (Li et al., 2018), enzyme catalysis powered mechanism (Lund et al., 2010), and photoinduced mechanism (You et al., 2012). While the successful assembly of the walker is often confirmed by TEM and gel electrophoresis, real-time observation of its locomotion and associated shape changes are imperative requirements. smFRET offers real-time conclusive evidence on such dynamical processes. Using diffusion-based smFRET-ALEX, Tomov et al. (2013) studied the stepping mechanism of non-autonomous bipedal DNA walker. Here, fuel strands connect the legs of the walker to the origami track, while antifuel strands release its legs from the track. smFRET-ALEX investigation quantitatively measured the kinetic rates of stepping by continuously monitoring the leg-lifted (associated with low *E*) and leg-placed (associated with high *E*) population among the DNA walkers. The 2D *E*/*S* histogram allowed the authors to separate the signals originating from tracks without a walker on them. The intact walker population on the tracks yielded distinctly different *S* values compared to tracks without any walker on them. Monitoring of the leg-lifted and leg-placed population with time revealed limited operational yield of the walker where only 1% of it remained intact on the track after few stepping cycles. Subsequent detailed kinetic studies led to a rational redesign of the antifuel strand, and the efficiency of the walker was dramatically improved. Furthermore, a recent report employed immobilization-based smFRET to demonstrate the rapid locomotion of a DNA walker by a cartwheeling mechanism (Li et al., 2018). The walker exploited toehold exchange mechanism for its locomotion. smFRET intensity trajectories directly reflected the effect of the toehold and branch migration domain length on stepping kinetics. The authors optimized the walker’s stepping rate by taking cues from the systematic smFRET investigation, thus designing one of the fastest moving DNA walkers reported so far. Concomitantly, Valero et al. (2018) reported another DNA walker which walks unidirectionally along a predefined track. A biohybrid interlocked rotor–stator unit fueled by the NTP hydrolysis by T7 RNA polymerase constituted the engine of the translational DNA walker. The interlocked rotor–stator unit was made of double-stranded DNA rings, and donor and acceptor fluorophores were placed on the rotor and stator, respectively. The unidirectional rotation of the biohybrid engine was confirmed by the TIRFM-based smFRET study. An oscillating FRET efficiency *E* was observed as the distance between the fluorophores changed periodically during rotation of the rotor with respect to the stator. The rotational speed of the rotor was directly measured from dwell times in the high and low FRET states. This was essential information in determining the DNA walker speed. Altogether, these studies demonstrated how smFRET can be useful in developing general design strategies for high-speed DNA walkers.

### Characterizing Drug Delivery Nanovehicle

Many recent studies highlight the potential application of DNA nanostructures as a drug delivery vehicle. Toward that goal, DNA nanostructures of all sizes and shapes have been designed. They have been decorated with therapeutic oligonucleotides (Li et al., 2011), small molecules (Zhang et al., 2014), antibodies (Douglas et al., 2012), etc. Dynamic features of these drug delivery vehicles allowed conformational changes and subsequent cargo delivery. One such example is a DNA nanobox with a controllable lid designed by Andersen et al. (2009). The nanobox has a large enough cavity to carry cargo. The authors dynamically controlled the opening and closing of the lid with short oligonucleotides as keys and monitored the two states with ensemble averaged FRET. However, the difference in FRET efficiencies *E* between the open and closed boxes was found to be smaller than expected. Interestingly, a recent smFRET study complemented by molecular dynamics (MD) simulation confirmed local distortion near the lid and its saggy closure, which accounted for the smaller difference in *E* between the two states of the box (Jepsen et al., 2019). Systematic smFRET studies under different ionic conditions along with altered design helped the authors to decrease the local fluctuation and achieve better closure of the lid. Such studies aided in improving the stability of the DNA nanostructure, an absolute requirement for its optimum function.

DNA tetrahedron is the most popular and one of the simplest DNA nanostructures for developing drug delivery vehicles. The drug-releasing mechanism of DNA tetrahedron is often initiated by reversible shape change in response to a molecular signal. Goodman et al. (2008) confirmed the sequential opening and closing of the tetrahedron by smFRET-ALEX using hairpin loops as a convertible structural motif. The FRET efficiency histograms reflected efficient interconversion of closed tetrahedra (high FRET efficiency) into open tetrahedra (high FRET efficiency). These studies pointed out the necessity of proper characterization of the DNA nanostructures before they are applied as intelligent drug delivery systems. However, despite having the potential to assist in designing and assessing the operational competence, the application of smFRET in studying DNA nanostructure-based drug delivery nanovehicle is limited.

### Characterizing DNA Tweezers

DNA tweezers are one prototype of DNA nanodevices that has been used for force measurement (Funke et al., 2016), investigating molecular interaction (Liu et al., 2013), and as a molecular logic gate (Li et al., 2016). However, to improve the performance of the DNA tweezers from suboptimal to expected high, if not ideal, performance, improved engineering of the structure is required. Additionally, a single conformation of the tweezers in each state (“open” state and “closed” state) is an essential requirement for standardization of the device. Identifying the importance of investigation at a single nanostructure level, Muller et al. (2006) reported the conformational heterogeneity in DNA tweezers from a diffusion-based single-pair FRET study. The study revealed the existence of three subpopulations of DNA tweezers in their “closed” form. Incomplete closure of the arms and undesired dimer formation accounted for the observed heterogeneity. For improved proximity between the arms of a tweezer in a closed state, Dhakal et al. (2016) leveraged TIRFM-based smFRET to systematically study the design strategy. The FRET pair attached at the two arms of the tweezers reported its conformational state. Although a single, zero-FRET population was observed for an open tweezer, the closure of the tweezer yielded two populations. A subset of the tweezers remained in the open state. All-atom MD simulation inspired sequence redesign for improved closure of tweezer arms and minimization of conformational heterogeneity. The success of the design strategy was confirmed from smFRET data. The optimum design strategy reduced the conformational heterogeneity among the tweezers and offered controlled inter-arm distance. These studies exemplify how a single-molecule (or single nanostructure) study is essential to gain insights into the structural diversity of even a seemingly simple nanodevice such as DNA tweezers. Likewise, a recent study reported actuation dynamics of ion-mediated DNA origami-based tweezers. The authors employed smFRET to monitor the repeated opening and closing of the arms in real time (Marras et al., 2018). These repeated conformational changes in the DNA nanodevices in response to change in ionic environment are reported to occur at the millisecond timescale.

### Study of Bioassay on DNA Nanostructures

Due to its addressability, DNA origami nanostructures offer us an ideal platform to arrange biomolecules with nanoscale precision (Fu et al., 2016). They not only provide control over the spatial distribution of the biomolecules but also help in maintaining the stoichiometry of the participating molecules. One such example is a substrate cascading assay assembled on a DNA origami platform (Fu et al., 2014).

The DNA double-strand break (DSB) is one of the most cytotoxic forms of DNA damage. A mis-repaired DSB can cause the onset of cancer progression. A recent study by Bartnik et al. (2020) employed smFRET to study DNA DSB repair mechanism by T4 DNA ligase on a DNA origami structure. Through smFRET signals, the authors directly monitored the dynamic ligation reaction. The study underpinned the suitability of the DNA origami platform to study complex biomolecular reactions. Similarly, DNA distortion by transcription factor TATA-binding protein (TBP) under tension was studied by assembling the complex on a DNA origami-based force clamp. smFRET signals were used as a direct readout for quantifying the DNA distortion by TBP under tension (Nickels et al., 2016). A high FRET population, reflective of TBP-induced DNA bending, gradually decreased with increasing force applied by DNA origami-based force clamp. Here, the DNA origami-based force clamp acted as a non-invasive manipulation tool. Altogether, these smFRET investigations established that DNA nanostructures can not only provide a static platform for organizing biomolecules but also act as a nano-actuator.

### Developing Biosensors

In nucleic acid-based biosensors, the sensing element often undergoes conformational change upon target binding. On the other hand, hybridization-based biosensors utilize selective hybridization of the target sequence to the single-stranded probe sequence. The hybridization kinetics is associated with its characteristic kinetic fingerprint contingent on the target and probe sequences. Such kinetic fingerprint alters in the presence of non-specific bindings. These sensors are often immobilized on 2D and 3D DNA origami matrices as DNA origami pegboards offer a platform for organizing ligands and chemical groups at precise locations with *nm* precision (Johnson-Buck et al., 2013; Fu et al., 2014). However, the steric hindrance from the matrix and inter-sensor proximity are important parameters to study while designing a sensor with optimum sensitivity. Such characterization is becoming increasingly crucial to understand the effect of the DNA origami matrix on hybridization-based nucleic acid detection techniques, for example, microRNA detection (Zhu et al., 2013; Wang et al., 2014). Johnson-Buck et al. (2013)have utilized the strength of smFRET to systemically study the association and dissociation kinetics of oligonucleotides on a 2D DNA origami platform. The study revealed significant impact of the array design on hybridization kinetics. The result is of paramount importance as it showed the potential impact of the DNA origami pegboard on the overall performance of DNA nanodevices. Additionally, such information can possibly account for the differences in performance among individual sensors. In a different study, Hemmig et al. (2018)designed a DNA origami-based voltage sensor where the smFRET signal is used as a direct readout for voltage. The potential application of such DNA origami-based system as a transmembrane voltage sensor has been extended by Ochmann et al. (2021). The programmability of DNA origami has enabled incorporation of both the membrane-targeting and sensing modules. The transmembrane voltage readout from the surface of a lipid membrane was defined by the smFRET signal.

These investigations reinforce the strength of smFRET as a tool to optimize DNA nanostructure-based sensors. These optimizations not only improve the performance but also minimize the undesired non-specific interactions.

## Conclusion and Future Outlooks

In this mini review, we have presented a consolidated summary on characterization of DNA nanostructures at single nanostructure level by using smFRET. It is becoming increasingly important to have quantitative information on the working mechanism of DNA nanostructure-based devices. Such information is essential to refine the design and improve stability and performance. Toward this purpose, smFRET-based techniques have been employed to study dynamic DNA nanostructures. These studies illustrated how smFRET characterization aided to explore the synergism between optimum design and optimum function of these DNA nanostructures and the devices they make. Despite having enormous potential, smFRET-based techniques have not been utilized to their full strength when it comes to characterizing DNA nanostructure-based machines. One of the reasons might be that these devices are often slow in response or sluggish in performance. This requires a long observation time window, which is often limited by the photobleaching of the fluorophore. Using an efficient oxygen scavenger system and alternating excitation of the fluorophores might ensure its longer survival. Moreover, integrating multiple techniques such as smFRET with AFM or tweezers can be advantageous. As an example, force manipulation using AFM or magnetic tweezers would be an interesting approach toward characterization of these DNA nanostructures in a complex environment. Such correlated measurements have the potential to unravel intricate details of mechanistic steps.
